# Structural, Elastic, Electronic, Dynamic, and Thermal Properties of SrAl_2_O_4_ with an Orthorhombic Structure Under Pressure

**DOI:** 10.3390/molecules29215192

**Published:** 2024-11-02

**Authors:** Hongli Guo, Huanyin Yang, Suihu Dang, Shunru Zhang, Haijun Hou

**Affiliations:** 1 College of Electronic and Information Engineering, Yangtze Normal University, Chongqing 408000, China; yuxinyin83@163.com (H.Y.); dangsuihu@126.com (S.D.); 2 School of Physics, Electronics and Intelligent Manufacturing, Huaihua University, Huaihua 418008, China; zefer1979@163.com; 3 School of Materials Engineering, Yancheng Institute of Technology, Yancheng 224051, China

**Keywords:** SrAl_2_O_4_, elastic constants, electronic, dynamic

## Abstract

Its outstanding mechanical and thermodynamic characteristics make SrAl_2_O_4_ a highly desirable ceramic material for high-temperature applications. However, the effects of elevated pressure on the structural and other properties of SrAl_2_O_4_ are still poorly understood. This study encompassed structural, elastic, electronic, dynamic, and thermal characteristics. Band structure calculations indicate that the direct band gap of SrAl_2_O_4_ is 4.54 eV. In addition, the Cauchy pressures provide evidence of the brittle characteristics of SrAl_2_O_4_. The mechanical and dynamic stability of SrAl_2_O_4_ is evident from the accurate determination of its elastic constants and phonon dispersion relations. In addition, a comprehensive analysis was conducted of the relationship between specific heat and entropy concerning temperature variations.

## 1. Introduction

Many scientific studies have been conducted to analyze aluminate compounds possessing the structure AB_2_O_4_, owing to their exceptional properties, such as outstanding reflectivity and excellent resistance to chemical degradation at elevated temperatures. Furthermore, these compounds exhibit minimal electrical loss [1,2]. In addition, aluminates demonstrate environmentally friendly characteristics, display enhanced chemical durability, and can be easily manufactured at a cost that is deemed reasonable [3]. Consequently, more research investigations are being conducted to explore the synthesis and examination of phosphors based on aluminate, and there is a growing interest in conducting research studies to investigate the synthesis and analysis of aluminate-based phosphors [4,5,6]. In various fields of application, such as light-emitting diodes and imaging devices, there is a demand for inorganic phosphorus materials that exhibit exceptional color clarity and intensity [7,8,9]. Extensive research has been conducted on AB_2_O_4_ substances that display an orthorhombic configuration reminiscent of CaFe_2_O_4_ [10,11,12,13,14,15,16]. In particular, SrAl_2_O_4_ exhibits remarkable luminescent properties. These materials possess versatile applications in diverse sectors, including but not limited to display technologies, signage solutions, medical advancements, and storage innovations. Therefore, it is important to highlight the research conducted on SrAl_2_O_4_. Nazarov and colleagues [10] utilized density functional theory to examine the modifications in both structural and electronic characteristics of SrAl_2_O_4_ following the introduction of Eu^3+^ doping. They discovered that the band gap of SrAl_2_O_4_, measured at 4.52 eV, is lower than the experimental value of 6.5 eV. In a separate investigation, Rojas-Hernandez et al. examined the structural characteristics of SrAl_2_O_4_’s P2_1_, P6_3_22, and P6_3_ phases utilizing both LDA and GGA techniques [14]. Recently, the hexagonal structure of XAl_2_O_4_ (X = Ca, Sr and Cd) has been investigated by Ref. [15]. In addition, research has been conducted on the structural, elastic, electronic, and vibrational properties of XAl_2_O_4_ (X = Ca, Sr and Cd) semiconductors with an orthorhombic structure [16].

However, there is currently a lack of comprehensive calculations available for orthorhombic SrAl_2_O_4_ under varying pressures. So, the primary objective of this study was to acquire a comprehensive understanding of the elastic, electronic, dynamic, and thermodynamic characteristics of SrAl_2_O_4_. Calculations based on density functional theory were utilized to achieve this objective. Pressure exerts an influence on both the lattice parameters and elastic constants of a substance, ultimately leading to alterations in its lattice parameters, elastic modulus, and melting point. Hence, it is imperative to investigate the impact of elevated pressure on the mechanical and thermodynamic characteristics of materials at high temperatures. The impact of elevated pressure on the structural, mechanical, and thermodynamic properties of SrAl_2_O_4_ has remained uncertain. To address that research gap, this study employed a first-principles approach to investigate how pressure influences the structural, elastic, electronic, dynamic, and thermal characteristics of SrAl_2_O_4_.

## 2. Results and Discussion

### 2.1. Structural Parameters

**Figure 1:**
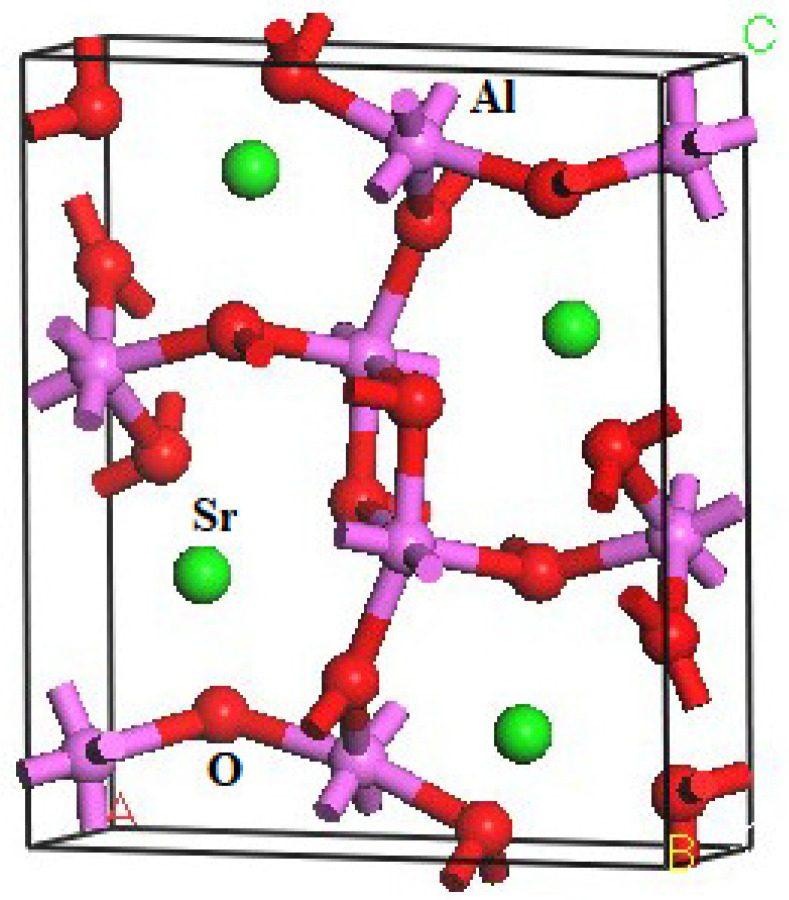
Crystal structure of orthorhombic SrAl_2_O_4_ (The green, purple, and red balls are Sr, Al, and O atoms, respectively).

The crystal structure of SrAl_2_O_4_ is characterized by a CaFe_2_O_4_-type arrangement (Pnma). The DFT method was employed to compute the structural parameters of orthorhombic SrAl_2_O_4_. The structural parameters of SrAl_2_O_4_, and the corresponding theoretical data [16], are presented in Table 1. Furthermore, the atomic coordinates at equilibrium for SrAl_2_O_4_ in the Pnma phase can be found in Table S1. The crystal structure of SrAl_2_O_4_, as depicted in Figure 1, is derived from the computed positional arrangements. The results are consistent with the theoretical information presented in reference [16].

### 2.2. Mechanical Properties

In order to assess the stability of SrAl_2_O_4_ and gain deeper insights into its pressure-induced anisotropic structural characteristics, we conducted elasticity calculations for this compound. In particular, calculations were performed to determine the elastic constants *C*_ij_. For crystals with an orthorhombic structure, the elastic constants must meet certain criteria to ensure mechanical stability [17].

(1) $$C_{11}>0,C_{44}>0,C_{55}>0,C_{66}>0$$

(2) $$C_{11}C_{12}>{C_{22}}^{2}$$

(3) $$C_{11}C_{22}C_{33}+2C_{12}C_{13}C_{23}-C_{11}{C_{23}}^{2}-C_{22}{C_{13}}^{2}-C_{33}{C_{12}}^{2}>0$$

The obtained effective elastic constants are provided in Table 2, along with the theoretical data at a pressure of 0 GPa. The obtained data align with the findings reported in Ref. [16]. In the pressure range of 0 to 50 GPa, it can be observed that the mechanical stability of the orthorhombic phase of SrAl_2_O_4_ is maintained, as evidenced by meeting the criteria for stability. In the subsequent stage, we employed the Reuss–Voigt–Hill approximation techniques to determine the bulk modulus *B*, shear modulus *G*, and Young’s modulus *E* of SrAl_2_O_4_ [18,19,20]. In Table 3, we have included the pressure-dependent values of *B*, *G*, and *E* for SrAl_2_O_4_. The values of *B*, *G*, and *E* at 0 K and 0 GPa are slightly higher than the theoretical predictions of 163.1 GPa, 101.09 GPa, and 256.28 GPa, respectively [16], with measured values of approximately 180.52 GPa, 108.31 GPa, and 270.77 GPa. Poisson’s ratio *v* can be judged as a criterion of brittleness/ductility. Poisson’s ratio *v* of SrAl_2_O_4_ remains consistently below 0.26 throughout a pressure range of 0–50 GPa, indicating its inherent brittleness. The ratio *B*/*G* has also been used to judge the brittleness/ductility of a solid [21]. The *B*/*G* ratio of SrAl_2_O_4_ is less than 1.75, indicating that SrAl_2_O_4_ is a brittle structure. Table 3 displays the values of Poisson’s ratio (*v*) and *B*/*G* for SrAl_2_O_4_ under zero pressure conditions. These values, 0.25 and 1.67, respectively, exhibit a remarkable agreement with the results obtained from other simulation methods, as shown in Table 3, where the corresponding values were 0.2381 and 1.58. The pressure-dependent variations in *v* and *B*/*G* for SrAl_2_O_4_ are presented in Table 3, indicating an upward trend with increasing pressure. The findings indicate that SrAl_2_O_4_ demonstrates a tendency towards brittleness within the pressure intervals of 0–50 GPa.

The anisotropic factors for shear on various crystallographic planes (*A*_1_, *A*_2_, and *A*_3_), the universal anisotropic index (*A^U^*), the percentage of anisotropy in shear and bulk moduli (*A_G_* and *A_B_*), as well as the directional bulk moduli (*B_a_*, *B_b_*, and *B_c_*) were computed and are summarized in Table 4. It is possible to assess the mechanical anisotropy indexes through the following methods [22,23,24]

*A*_1_ = 4*C*_44_/(*C*_11_ + *C*_33_ − 2*C*_13_) (4)

*A*_2_ = 4*C*_55_/(*C*_22_ + *C*_33_ − 2*C*_23_) (5)

*A*_3_ = 4*C*_66_/(*C*_11_ + *C*_22_ − 2*C*_12_) (6)

The orthorhombic crystal’s elastic anisotropy can be characterized by the bulk moduli *B_a_*, *B_b_*, and *B_c_* along the *a*, *b*, and *c* axes, respectively. These values are interrelated.

(7) $$B_{a}=a\frac{dP}{da}=\frac{\Lambda }{1+\alpha +\beta }$$

(8) $$B_{b}=b\frac{dP}{db}=\frac{B_{\alpha }}{\alpha }$$

(9) $$B_{c}=c\frac{dP}{dc}=\frac{B_{\alpha }}{\beta }$$

where

$$\Lambda =C_{11}+2C_{12}\alpha +C_{22}\alpha^{2}+2C_{13}\beta +C_{33}\beta^{2}+2C_{23}\alpha \beta$$

$$\alpha =\frac{\left(C_{11}-C_{12})(C_{33}-C_{13})-(C_{23}-C_{13})(C_{11}-C_{13}\right)}{\left(C_{33}-C_{13})(C_{22}-C_{12})-(C_{13}-C_{23})(C_{12}-C_{23}\right)}$$

$$\beta =\frac{\left(C_{22}-C_{12})(C_{11}-C_{13})-(C_{11}-C_{12})(C_{23}-C_{12}\right)}{\left(C_{22}-C_{12})(C_{33}-C_{13})-(C_{12}-C_{23})(C_{13}-C_{23}\right)}$$

The anisotropy factor percentage for the orthorhombic crystal, derived from the bulk modulus (*A_B_*) and shear modulus (*A_G_*), can be determined using the equation proposed by Chung and Buessem [22,23,24].

(10) $$A^{U}=5\frac{G_{V}}{G_{R}}+\frac{B_{V}}{B_{R}}-6,$$

(11) $$A_{B}=\frac{B_{V}-B_{R}}{B_{V}+B_{R}},$$

(12) $$A_{G}=\frac{G_{V}-G_{R}}{G_{V}+G_{R}}$$

Table 4 presents the calculated values of *A*_1_, *A*_2_, and *A*_3_, indicating a deviation from unity. This suggests that SrAl_2_O_4_ demonstrates a significant elastic anisotropy, reaching up to 50 GPa. Meanwhile, the *a*-axis exhibits the highest directional bulk modulus for *B_a_* compared to *B_b_* and *B_c_*. The observed trend indicates that the compressibility of SrAl_2_O_4_ is comparatively higher along the *a* and *c* axes, while it exhibits relatively lower compressibility along the *b* axis. Additionally, it can be noted that compression along the *c* axis is relatively more favorable. This observation is consistent with our previous findings in Figure S1, indicating that the lattice parameter *b* exhibits a more rapid decrease under pressure than parameters *a* and *c*. If the value of *A^U^* is equal to zero, it indicates that the material exhibits isotropic properties. Otherwise, a greater departure from zero results in increased anisotropic elasticity. The range of *A_B_* and *A_G_* values spans from zero to one, denoting the presence of elastic anisotropy in terms of compression and shear characteristics, respectively. In Table 4, we additionally present the pressure-dependent anisotropic indexes. The anisotropy of SrAl_2_O_4_ is more pronounced within the pressure range of 0–50 GPa, leading to higher *A^U^* values. The *A^U^* of SrAl_2_O_4_ exhibits a continuous increase until reaching 50 GPa.

The Debye temperature (*θ*) has a close correlation with both the specific heat and melting temperature. Acoustic vibrations solely contribute to the crystal vibrations in conditions of low temperatures. Hence, by utilizing the elastic constants as input parameters, it is possible to determine the Debye temperature at low temperatures through established relationships [25]

(13) $$\theta =\frac{h}{k_{B}}{\left[\frac{3n}{4\pi }(\frac{NA\rho }{M})\right]}^{1/3}v_{m}$$

The given formula involves the utilization of various constants such as *ħ*, *k*, and *N_A_*, and variables including *n*, *M*, *ρ*, and *v_m_*. These elements are employed to calculate the average sound velocity in a manner that accounts for Planck’s constant divided by 2π, Boltzmann’s constant, Avogadro’s number, the number of atoms per formula unit, the molecular mass per formula unit, density, and the average sound velocity [26]

(14) $$v_{m}={\left[\frac{1}{3}(\frac{2}{v_{t}^{3}}+\frac{1}{{v_{l}}^{3}})\right]}^{-1/3}$$

The calculation of longitudinal and transverse elastic wave velocities involves utilizing the following relationship, where *v_t_* represents the longitudinal velocity and *v_l_* denotes the transverse velocity [27,28]

(15) $$v_{t}={\left(\frac{G}{\rho }\right)}^{1/2}$$

(16) $$v_{l}={\left[(B+\frac{4G}{3})/\rho \right]}^{1/2}$$

The values for the longitudinal, transverse, and average sound velocities and Debye temperature of SrAl_2_O_4_ under different pressures are presented in Table 3, alongside the findings reported in ref. [16]. After conducting a thorough comparison, it can be concluded that our calculated outcomes align well with existing research findings, affirming the accuracy of our calculations. According to the data provided in Table 3, it can be deduced that an upward trend exists between pressure and the longitudinal, transverse, and average sound velocities, as well as the Debye temperature.

In addition, the hardness *H_V_*, melting point *T*_m_, and thermal conductivity *k* of the material are calculated utilizing the subsequent formulas [29,30]

(17) $$H_{V}=0.92{\left(\frac{G}{B}\right)}^{1.137}G^{0.708}$$

*T*_m_ = 354 + 4.5(2*C*_11_ + *C*_33_)/3 (18)

(19) $$\kappa_{\min}=0.87k_{B}Ma^{-\frac{2}{3}}\rho^{\frac{1}{6}}E^{\frac{1}{2}}$$

Here, *k_B_* is Boltzmann’s constant, *ρ* is the density, and *M_a_* is the average mass per atom, respectively. At zero pressure, the material exhibits a hardness value of 14.2 GPa, a melting point temperature of 1646.10 K, and a thermal conductivity rate of 1.92 W·m^−1^·K^−1^. It is evident from the data presented in Table 4 that under increased pressure, *H_v_*, *T*_m_, and *k* exhibit noticeable increments.

**Figure 2:**
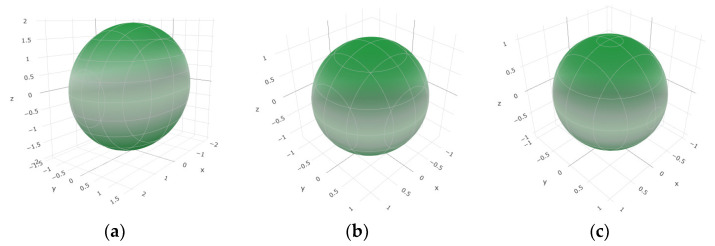
The surface contours of linear compressibility *β* (TPa^−1^) of SrAl_2_O_4_. (**a**) 0 GPa, (**b**) 30 GPa, (**c**) 50 GPa.

**Figure 3:**
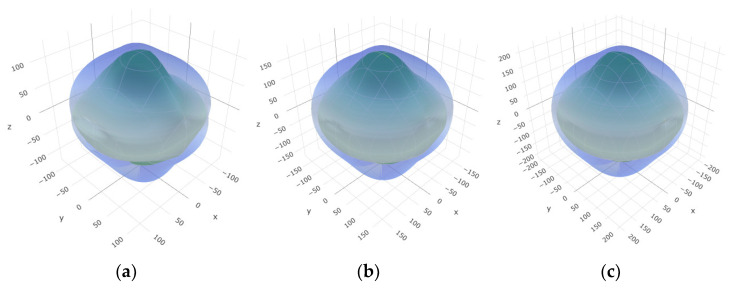
The surface contours of shear modulus *G* (GPa) of SrAl_2_O_4_. (**a**) 0 GPa, (**b**) 30 GPa, (**c**) 50 GPa.

**Figure 4:**
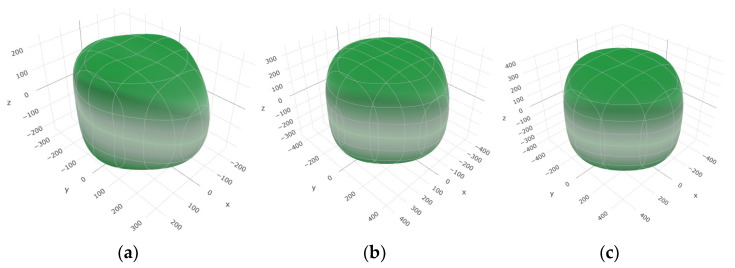
The surface contours of Young’s modulus *E* (GPa) of SAl_2_O_4_. (**a**) 0 GPa, (**b**) 30 GPa, (**c**) 50 GPa.

**Figure 5:**
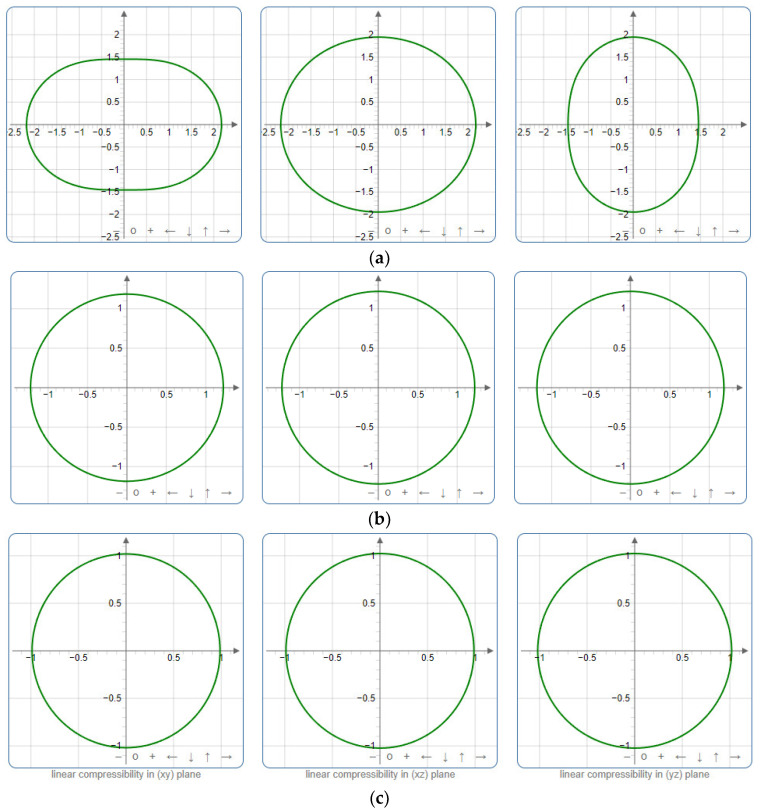
Projections of linear compressibility *β* (TPa^−1^) in xy, xz, and yz planes of SrAl_2_O_4_. (**a**) 0 GPa, (**b**) 30 GPa, (**c**) 50 GPa.

**Figure 6:**
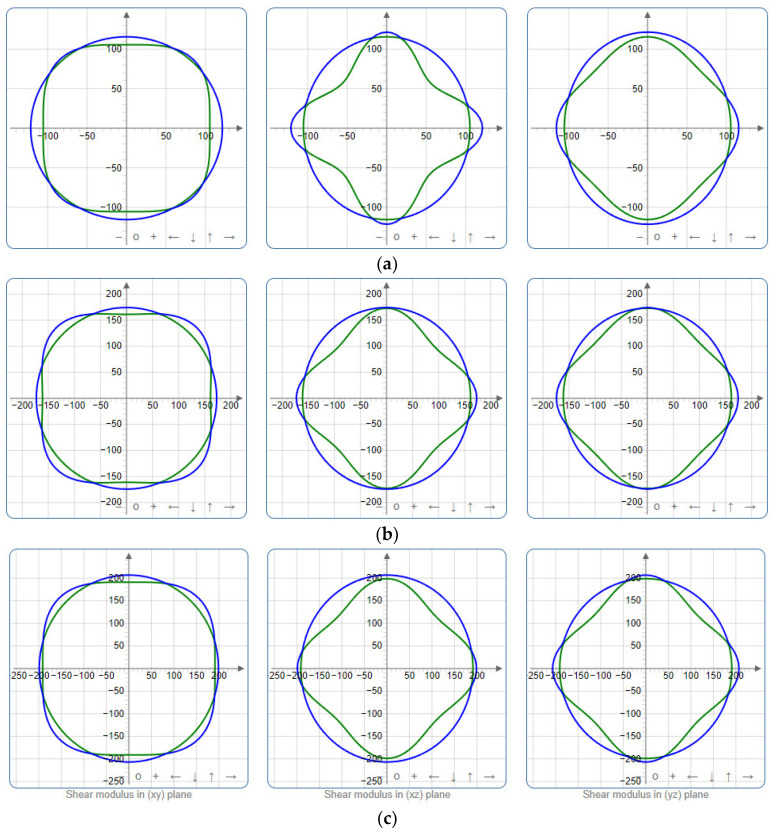
Projections of shear modulus *G* (GPa) in xy, xz, and yz planes of SrAl_2_O_4_. (**a**) 0 GPa, (**b**) 30 GPa, (**c**) 50 GPa.

**Figure 7:**
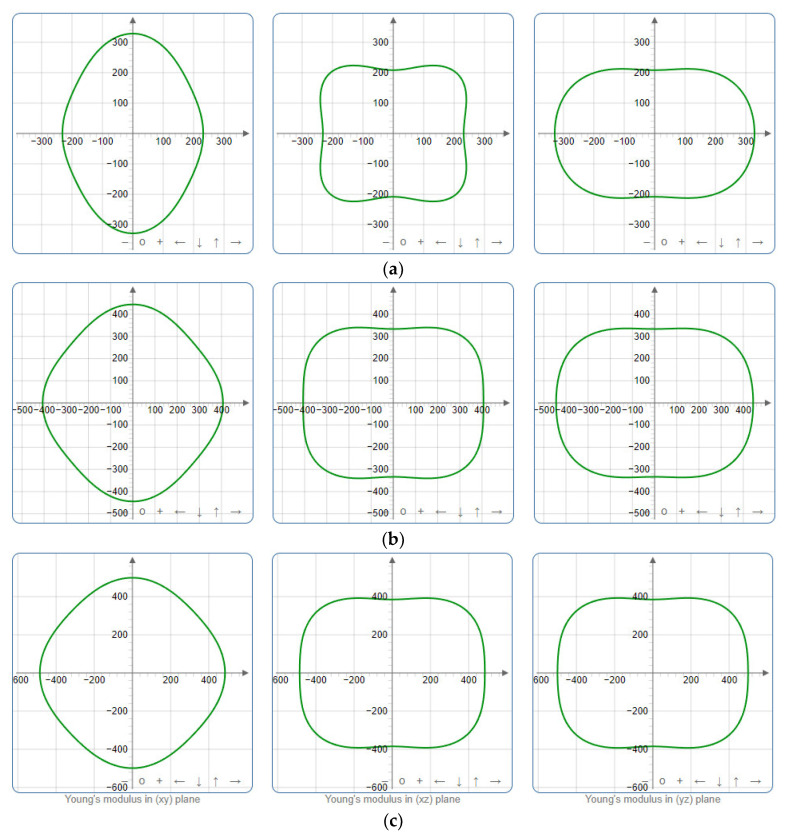
Projections of Young’s modulus *G* (GPa) in xy, xz, and yz planes of SrAl_2_O_4_. (**a**) 0 GPa, (**b**) 30 GPa, (**c**) 50 GPa.

To provide a comprehensive analysis of the mechanical anisotropy, a study was conducted on the spatial variation in linear compressibility (*β*), shear modulus (*G*), and Young’s modulus (*E*) in SrAl_2_O_4_ at varying pressure levels [31]. Figure 2, Figure 3 and Figure 4 illustrate the spatial correlation between linear compressibility (*β*), shear modulus (*G*), and Young’s modulus (*E*) in SrAl_2_O_4_. In the event of a structure exhibiting isotropy, the spatial correlation will be evident in a spherical configuration, whereby any deviation from this particular shape can be utilized as an indicator for identifying anisotropy. The non-spherical spatial distribution of linear compressibility (*β*), shear modulus (*G*), and Young’s modulus (*E*) at varying pressures in SrAl_2_O_4_ suggests a significant level of material anisotropy. For a more extensive view of the directional responsiveness of SrAl_2_O_4_’s linear compressibility (*β*), shear modulus (*G*), and Young’s modulus (*E*) under varying pressure conditions, Figure 5, Figure 6 and Figure 7 present the planar projections of linear compressibility (*β*), shear modulus (*G*), and Young’s modulus (*E*) in the xy, yz, and xz planes. For materials with properties that are the same in all directions, the curve of projection displays a circular form. For the xy and xz planes, there is a noticeable increase in deviation for the linear compressibility (*β*), shear modulus (*G*), and Young’s modulus (*E*), as shown in Figure 5, Figure 6 and Figure 7. This indicates a significant variation in elastic properties between the xy and xz planes for these solids. It suggests a notable disparity in the elastic characteristics between the xy and xz orientations within these materials. Simultaneously, the elastic anisotropy of SrAl_2_O_4_ was thoroughly examined under different pressure conditions by employing *β*_max_/*β*_min_, *G*_max_/*G*_min_, and *E*_max_/*E*_min_ ratios. Higher linear compression anisotropy can be inferred from a greater *β*_max_/*β*_min_ and the corresponding *G*_max_/*G*_min_ and *E*_max_/*E*_min_ ratios. The *β*_max_/*β*_min_ values are 1.50, 1.18, 1.08, 1.03, 1.02, and 1.04 at 0 GPa, 10 GPa, 20 GPa, 30 GPa, 40 GPa, and 50 GPa, respectively; the *G*_max_/*G*_min_ values are 1.59, 1.48, 1.44, 1.43, 1.41, and 1.41 at 0 GPa, 10 GPa, 20 GPa, 30 GPa, 40 GPa, and 50 GPa, respectively; and the *E*_max_/*E*_min_ values are 1.58, 1.43, 1.37, 1.35, 1.35, and 1.35 at 0 GPa, 10 GPa, 20 GPa, 30 GPa, 40 GPa, and 50 GPa, respectively. According to the data provided, anisotropy decreases with increasing pressure within the range of 0–50 GPa.

### 2.3. Electronic Properties

**Figure 8:**
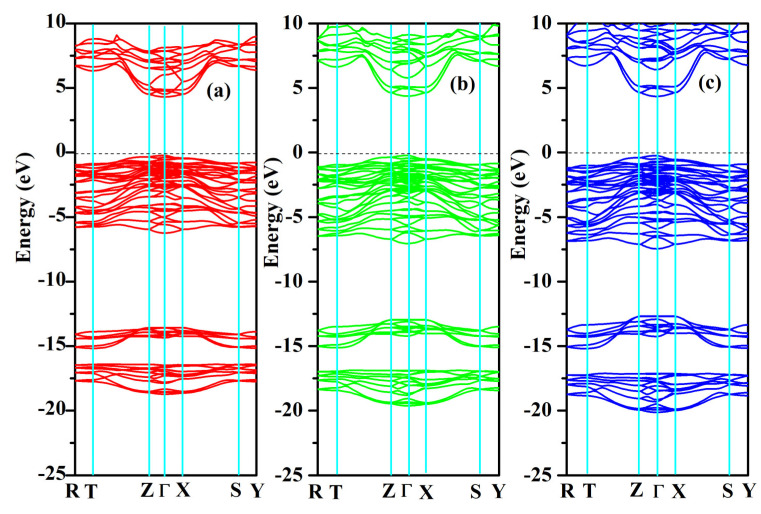
Electronic band structures for SrAl_2_O_4_. (**a**) 0 GPa, (**b**) 30 GPa, and (**c**) 50 GPa.

**Figure 9:**
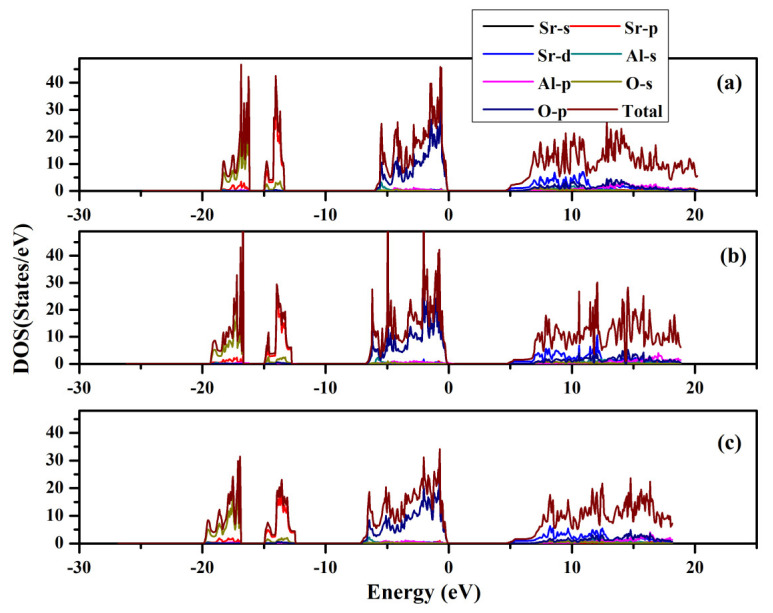
Total and partial density of states for SrAl_2_O_4_. (**a**) 0 GPa, (**b**) 30 GPa, and (**c**) 50 GPa.

The band structures and partial and total density of states were computed for SrAl_2_O_4_. Graphs illustrating these properties along high symmetry directions can be observed in Figure 8 and Figure 9. From these figures, it can be seen that there is a direct band gap of 4.54 eV for SrAl_2_O_4_. Our calculated band gap value for SrAl_2_O_4_ aligns well with the band gap (4.54 eV) reported in ref. [16]. Based on the analysis of the total and partial density of states for SrAl_2_O_4_ (Figure 9), it can be inferred that the primary contribution to the valence band of SrAl_2_O_4_ comes from the p orbitals of O atoms, with minor contributions from both the d and p orbitals of strontium atoms, as well as the p orbitals of Al atoms. Moreover, the remaining orbitals of the constituent atoms make negligible contributions. Based on the observations in Figure 9, it can be noted that there is an increase in the involvement of the O atom’s p orbitals as we progress from the lower region of the valence band toward the Fermi surface. The primary composition of the conduction band in SrAl_2_O_4_ is attributed to the d orbitals of Sr atoms, with minor involvement from the p orbitals of O atoms and negligible contributions from other constituent atom orbitals.

### 2.4. Phonon Properties

**Figure 10:**
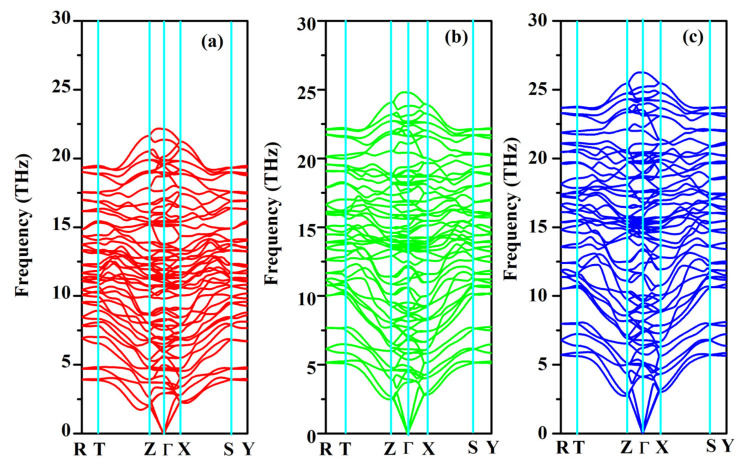
Phonon band structure for SrAl_2_O_4_. (**a**) 0 GPa, (**b**) 30 GPa, and (**c**) 50 GPa.

**Figure 11:**
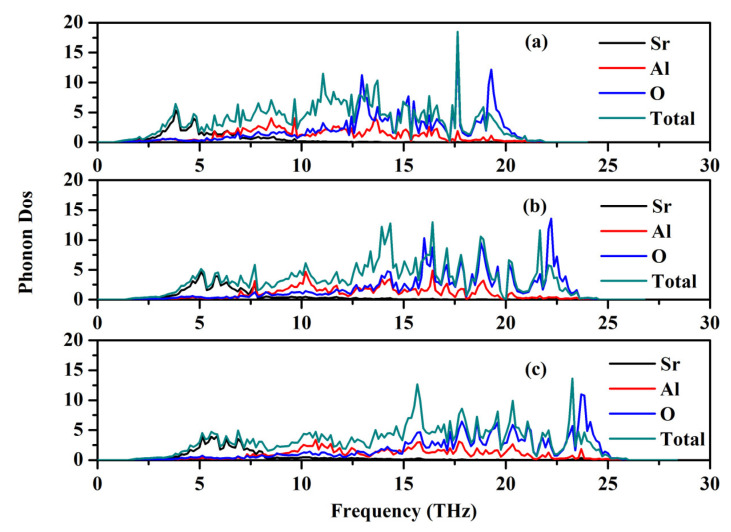
Total and partial phonon density of states (PDOS) for SrAl_2_O_4_. (**a**) 0 GPa, (**b**) 30 GPa, and (**c**) 50 GPa.

The phonon dispersion relation and the total and partial phonon density of states for SrAl_2_O_4_ in its orthorhombic structure are depicted in Figure 10 and Figure 11, respectively. The dispersion relation curves indicate the absence of any gap between the overall spectra, and no negative phonon frequencies can be observed. Hence, our findings suggest that the investigated SrAl_2_O_4_ exhibits favorable dynamic stability. According to the results depicted in Figure 10, the phonon dispersion curves of SrAl_2_O_4_ exhibit similarities to those observed at 0 GPa, even under increased pressures of 30 GPa and 50 GPa. As the pressure rises, there is a shift in the phonon frequency towards higher energy levels. It is evident that the low energy region depicted in Figure 11a primarily exhibits vibrations within the frequency range of 0–7.5 THz, predominantly originating from Sr atoms with a minor contribution from Al and O atoms. As the frequency rises, the influence of Sr atom vibrations diminishes while the impact of O and Al atom vibrations amplifies. In the mid-frequency range of 7.5–15.0 THz, the dominant vibrations are attributed to O and Al atoms, while the involvement of Sr atom vibrations diminishes. In the higher frequency range of 15.0–22.5 THz, the dominant vibrations are primarily attributed to the vibrational contributions from O and Al atoms, while the involvement of Sr atom vibrations is minimal in this high energy region. When examining Figure 11b,c, it becomes apparent that the vibration frequencies of Sr atoms, O atoms, and Al atoms progressively shift towards higher values as the applied pressure increases. In the low frequency range, there is a reduction in the vibrational contribution of Sr atoms as pressure levels increase.

The Pnma space group is associated with the orthorhombic crystal system of SrAl_2_O_4_. The irreducible representation of crystals with the Pnma structure in the center of the Brillouin zone (*k* = 0) is as follows:

*Γ* = 13A_g_ + 13B_2g_ + 13B_1u_ + 13B_3u_ + 8A_u_ + 8B_1g_ + 8B_3g_ + 8B_2u_ (20)

The Raman activity is observed in the 13A_g_, 8B_1g_, 13B_2g_, and 8B_3g_ modes, while the infrared activity is detected in the 12B_1u_, 7B_2u_, and 12B_3u_ modes. One B_1u_, one B_2u_, and one B_3u_ mode exhibit acoustic characteristics, whereas the 8A_u_ mode does not display Raman or infrared activity. Consequently, the Au mode can be classified as an acoustic mode. Table 5, Table 6 and Table 7 present the phonon frequencies at the *Γ* point and other computed values for SrAl_2_O_4_ under varying pressure conditions. In contrast, the computed values obtained under zero pressure concur with alternative theoretical findings [16].

**Figure 12:**
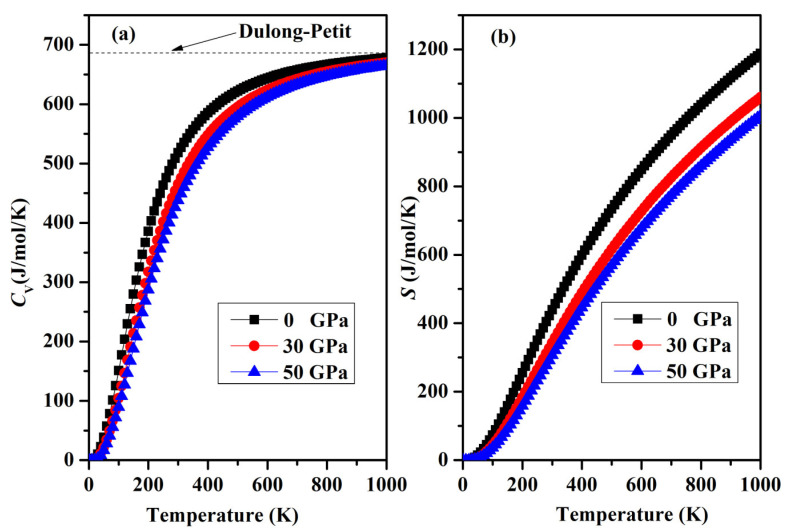
(**a**) Variation in the specific heat capacity *C*_V_ and (**b**) entropy *S* for orthorhombic SrAl_2_O_4_.

Finally, a graphical representation of the temperature-dependent thermodynamic properties, including specific heat *C*_V_ and entropy *S*, for SrAl_2_O_4_ can be observed in Figure 12a,b. As the temperature increases, there is a rapid increase in heat capacity until it reaches approximately 400 K. Beyond this temperature, the rate of increase becomes smaller. The heat capacity reaches a stable value called the Dulong–Petit limit when temperatures are elevated. In addition, the increase in temperature shown in Figure 12b is accompanied by an increase in entropy. Regrettably, there is a lack of empirical and theoretical information available for the comparison of thermodynamic properties.

## 3. Computational Methods

**Figure 13:**
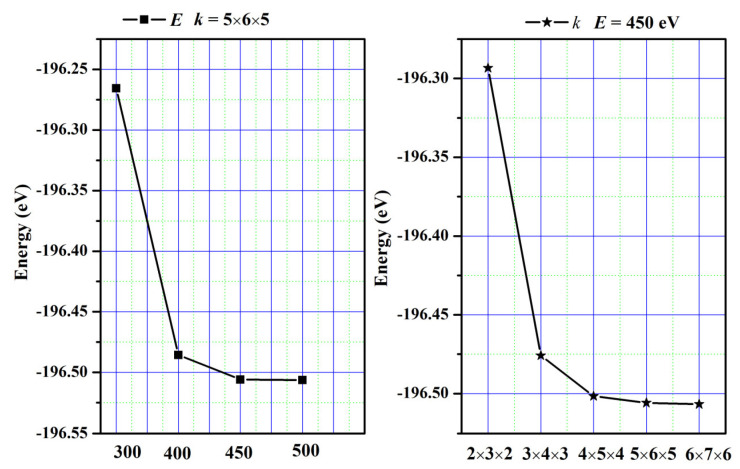
The convergence curves.

Relevant calculations were conducted utilizing the VASP code for the first principles of density functional theory [32,33]. To perform computations, we utilize Projected Augmented Wave (PAW) pseudo-potentials [34] in conjunction with the Generalized Gradient Approximation developed by Perdew et al. (GGA-PBE) [35]. The integration of the Brillouin zone was achieved by employing Monkhorst–Pack-generated sets of *k*-points. For SrAl_2_O_4_, a grid of 5 × 6 × 5 *k*-points and a kinetic energy cutoff value of 450 eV were used to achieve the desired level of convergence. The convergence curves are plotted in Figure 13. To solve the Kohn–Sham equation, an energy tolerance of 10^−4^ eV/cell was selected, and a force tolerance of 0.001 eV/Å was chosen to minimize the Hellman–Feynman force. The valence electron configurations were as follows: Sr atom is 4s^2^p^6^5s^2^, Al atom is 3s^2^p^1^, and O atom is 2s^2^p^4^. Moreover, phonon properties were investigated using the phonopy code [36].

## 4. Conclusions

In this study, we employed the first-principles method to investigate the effects of pressure on the structural, elastic, electronic, and dynamic properties of orthorhombic SrAl_2_O_4_. The elastic constants acquired indicate that SrAl_2_O_4_ possesses mechanical stability as they adhere to the criteria established by Born–Huang. Moreover, the Cauchy pressures indicate that the SrAl_2_O_4_ tends towards brittleness. The compressibility of SrAl_2_O_4_ is higher along the *a* and *c* axes, while it exhibits lower compressibility along the *b* axis. The elastic properties of SrAl_2_O_4_ are anisotropic. Finally, this study examined the dynamic characteristics and confirmed that the phonon dispersion curves of SrAl_2_O_4_ demonstrate dynamic stability. An investigation of the thermodynamic characteristics of SrAl_2_O_4_ was conducted as well. We noted that as the temperature increases, the specific heat values tend to approach the Dulong–Petit limit.

## Figures and Tables

**Table 1:** Calculated lattice parameters (*a*, *b*, *c*) (Å), density *ρ* (g/cm^3^), and volume *V* (Å^3^) of SrAl_2_O_4_.

	*a*	*b*	*c*	*ρ*	*V*
This work 0	9.30	2.94	10.67	4.69	291.39
Ref. [16] 0	9.326	2.941	10.696	4.655	293.349
10	9.13	2.90	10.49	4.93	277.19
20	8.99	2.86	10.34	5.14	265.85
30	8.88	2.83	10.22	5.32	256.47
40	8.79	2.80	10.11	5.50	248.21
50	8.70	2.77	10.01	5.66	241.30

**Table 2:** The elastic constants *C*_ij_ (GPa) of SrAl_2_O_4_ under different pressures.

Pressure	*C*_11_	*C*_12_	*C*_13_	*C*_22_	*C*_23_	*C*_33_	*C*_44_	*C*_55_	*C*_66_
0	289.62	88.73	124.14	388.38	124.55	282.23	115.65	121.65	105.58
0 Ref. [16]	258.0	72.93	117.97	370.91	101.99	262.92	115.32	113.52	100.99
10	376.58	98.84	151.76	437.34	142.34	349.14	136.58	141.57	125.84
20	441.89	112.65	174.70	482.32	161.94	401.25	156.24	158.26	144.42
30	498.18	127.85	196.15	524.26	182.04	446.08	174.27	172.98	161.20
40	550.71	144.04	217.13	564.22	202.62	487.01	191.14	186.50	176.72
50	599.81	160.80	237.96	601.16	223.34	525.14	206.94	198.95	191.14

**Table 3:** Calculated bulk modulus *B* (GPa), shear modulus *G* (GPa), Young modulus *E* (GPa), *B*/*G*, Poisson’s ratio *ν*, wave velocities *v*l, *v*t, and *v*m (km/s), and Debye temperatures *θ* (K) of SrAl_2_O_4_ under pressures ranging from 0 to 50 GPa.

Pressure (GPa)	*B*	*G*	*E*	*B/G*	*ν*	*v*_l_	*v*_t_	*v*_m_	*θ*
0	180.52	108.31	270.77	1.67	0.25	8.33	4.81	5.34	727.51
Ref. [16]	163.1	101.09	256.28	1.58	0.2381	8.039	4.71	5.225	711.2
10	216.29	130.62	326.20	1.666	0.25	8.90	5.15	5.72	792.17
20	247.03	148.72	371.59	1.66	0.25	9.31	5.38	5.97	839.47
30	275.61	164.33	411.26	1.68	0.25	9.64	5.56	6.17	877.35
40	303.25	178.50	447.67	1.70	0.25	9.92	5.70	6.33	909.78
50	330.02	191.21	480.77	1.73	0.26	10.17	5.81	6.46	937.53

**Table 4:** The shear anisotropy factors *A*_1_, *A*_2_, *A*_3_, the directional bulk modulus *B_a_*, *B_b_*, and *B_c_*, the elastic anisotropy index *A^U^*, *A_B_*, and *A_G_*, hardness *H_v_* (GPa), the melting temperature *T*_m_ (K), and thermal conductivity *k* (W·m^−1^·K^−1^) of SrAl_2_O_4_ under pressures ranging from 0 to 50 GPa.

Pressure	*A*_1_	*A*_2_	*A*_3_	*B_a_*	*B_b_*	*B_c_*	*A^U^*	*A_B_*	*A_G_*	*H_V_*	*T*_m_	*k*
0	1.43	1.15	0.84	460.05	687.54	513.86	0.16	0.60	1.17	14.19	1646.10	1.92
0 Ref. [16]	1.6186	1.0564	0.8363	386.25	766.0	477	0.2508	0.6625	2.3194			
10	1.29	1.13	0.82	604.92	713.85	635.25	0.11	0.11	1.15	16.33	2007.45	2.12
20	1.27	1.13	0.83	716.94	775.41	732.74	0.10	0.03	0.99	17.83	2281.50	2.28
30	1.26	1.14	0.84	817.99	843.35	819.53	0.09	0.0036	0.93	18.93	2517.75	2.41
40	1.27	1.16	0.86	916.19	913.44	899.78	0.09	0.0016	0.92	19.78	2736.60	2.53
50	1.28	1.17	0.87	1012.13	981.33	977.28	0.09	0.0045	0.94	20.40	2941.05	2.63

**Table 5:** Phonon frequencies (THz) at the *Γ* point for orthorhombic SrAl_2_O_4_ at 0 GPa.

Modes	Present	Theo. [16]	Modes	Present	Theo. [16]	Modes	Present	Theo. [16]	Modes	Present	Theo. [16]
A_u_	2.93	2.993	B_1u_	7.80	7.973	A_g_	11.50	11.541	A_g_	15.76	15.744
B_1u_	2.93	2.671	A_g_	7.87	7.611	B_1u_	11.53	11.417	B_3u_	15.94	15.886
B_3u_	3.31	3.282	B_3u_	8.18	8.012	B_1g_	11.54	11.637	B_1u_	16.05	16.101
B_3g_	3.70	3.559	B_1g_	8.32	8.502	B_2g_	11.89	11.872	B_2g_	16.50	16.089
B_1g_	3.71	3.563	A_g_	8.82	8.601	B_3u_	11.93	11.872	A_g_	16.97	16.934
A_g_	4.19	4.180	B_3u_	8.99	8.745	A_g_	12.36	12.461	B_2g_	17.11	17.010
A_u_	4.60	4.990	B_1u_	9.04	8.796	B_3u_	12.60	12.509	A_g_	17.66	17.593
A_g_	4.62	4.592	B_2g_	9.15	8.884	A_u_	12.60	12.769	B_3u_	18.69	18.585
B_2u_	4.76	4.744	A_g_	9.81	9.431	B_2u_	12.61	12.495	B_1u_	18.82	18.652
B_2g_	4.99	4.787	B_1u_	10.32	10.235	B_2g_	12.80	12.938	B_3u_	18.99	18.988
A_u_	5.53	6.035	B_3u_	10.44	10.115	B_1u_	13.25	13.432	B1u	19.48	19.400
B_2g_	5.64	5.549	A_u_	10.68	11.166	A_u_	13.32	13.437	B_2g_	19.48	19.028
B_3u_	5.91	5.560	B_2u_	10.69	11.188	B_2u_	13.35	13.715	A_g_	19.61	19.449
B_1g_	6.03	6.483	B_2g_	10.70	10.619	B_3u_	13.53	13.461	A_g_	19.82	19.661
B_2u_	6.19	6.539	B_1g_	10.72	11.163	B_1u_	13.75	13.889	B_2g_	19.91	19.616
B_1u_	6.28	5.631	B_3g_	10.79	11.148	A_g_	13.76	15.029	B_2g_	20.13	19.736
B_3u_	6.51	6.325	A_g_	10.99	10.812	B_1g_	15.08	15.122	B_1u_	21.37	21.239
B_2g_	6.82	6.467	B_2g_	11.28	11.176	B_2g_	15.19	15.504	B_3u_	22.14	21.708
B_3g_	6.97	7.287	B_2u_	11.28	11.332	B_3g_	15.22	15.207			
B_1u_	6.97	6.812	A_u_	11.36	11.402	B_3g_	15.37	15.539			
B_3g_	7.54	7.847	B_3g_	11.47	11.605	B_1g_	15.46				

**Table 6:** Phonon frequencies (THz) at the *Γ* point for orthorhombic SrAl_2_O_4_ at 30 GPa.

Modes	Present	Modes	Present	Modes	Present	Modes	Present
A_g_	4.90	B_3u_	8.60	A_u_	13.682	B_1g_	18.72
B_3u_	3.94	B_1u_	8.54	B_2u_	13.760	B_1u_	21.62
A_u_	3.72	B_1u_	10.60	B_2g_	14.237	B_3u_	18.26
B_1u_	4.50	B_1u_	12.06	B_1g_	14.012	A_g_	19.14
A_u_	5.82	B_2g_	10.91	B_3g_	13.829	B_3u_	21.07
B_1g_	4.64	B_3u_	10.61	B_1u_	15.670	A_g_	17.79
B_3u_	7.01	B_2g_	13.25	A_g_	14.904	B_2g_	19.37
B_3g_	4.55	B_1u_	12.54	B_3u_	15.104	B_1u_	22.07
A_g_	5.39	A_g_	11.31	B_1u_	16.911	B_1u_	23.82
B_2u_	6.13	A_g_	13.18	B_2g_	15.608	A_g_	20.42
B_2g_	5.85	B_3u_	12.19	B_2u_	15.857	A_g_	22.57
B_1g_	7.14	A_g_	13.44	A_u_	15.873	B_2g_	22.34
B_3g_	8.15	B_2g_	13.44	A_g_	16.151	B_3u_	21.65
A_u_	6.79	B_3u_	13.54	B_2g_	17.540	B_2g_	22.68
B_1u_	7.74	B_2u_	13.32	B_1g_	18.063	B_2u_	16.91
B_2g_	6.91	A_g_	14.36	A_u_	16.868	A_g_	22.68
B_2u_	7.56	B_1u_	14.69	B_3g_	18.188	B_3u_	24.80
B_3g_	8.73	A_u_	13.52	B_2g_	19.115	B_2g_	23.14
B_1g_	9.52	B_3u_	14.41	B_1u_	18.201		
B_2g_	8.88	B_3g_	13.69	B_3u_	16.628		
A_g_	9.13	B_1g_	13.56	B_3g_	18.667		

**Table 7:** Phonon frequencies (THz) at the *Γ* point for orthorhombic SrAl_2_O_4_ at 50 GPa.

Modes	Present	Modes	Present	Modes	Present	Modes	Present
A_g_	5.16	B_3u_	9.47	B_1g_	15.03	B_3g_	20.37
B_3u_	4.12	B_1u_	9.23	B_1g_	15.46	B_1g_	20.43
A_u_	4.15	B_1u_	11.66	B_2u_	15.26	B_3u_	19.82
B_1u_	5.09	B_3u_	11.57	B_1u_	16.31	B_1u_	19.62
B_1g_	5.08	B_2g_	11.64	A_g_	16.46	A_g_	20.47
B_3u_	7.34	B_2g_	14.25	B_3g_	15.32	A_g_	22.02
A_u_	6.42	A_g_	12.40	B_3u_	16.56	B_2g_	21.08
B_3g_	4.97	A_g_	14.63	B_1u_	16.80	B_3u_	22.27
A_g_	5.78	B_1u_	13.30	B_2g_	17.08	B_1u_	23.11
B_2g_	6.21	A_g_	14.55	B_2g_	18.72	B_1u_	23.53
B_2u_	6.78	B_2g_	14.56	A_g_	17.63	B_2g_	23.93
B_1g_	7.68	B_3u_	13.42	B_2u_	17.56	A_g_	24.22
B_3g_	8.73	A_g_	15.74	A_u_	17.60	B_3u_	23.43
B_1u_	8.15	B_3u_	14.93	A_g_	18.95	B_2g_	24.25
A_u_	7.45	B_2u_	14.63	B_1g_	19.64	B_1u_	25.19
B_2g_	7.49	B_1u_	13.82	A_u_	18.65	B_3u_	26.26
B_3g_	9.24	B_2g_	15.61	B_2g_	20.25	A_g_	24.37
A_g_	9.63	A_u_	14.96	B_2u_	18.71	B_2g_	24.87
B_2u_	8.22	B_3g_	15.09	B_3g_	19.78		
B_1g_	10.05	B_3u_	15.66	B_1u_	18.95		
B_2g_	9.73	A_u_	15.06	B_3u_	18.60		

## Data Availability

Data are contained within the article and Supplementary Materials.

## Supplementary Materials

The following supporting information can be downloaded at: https://www.mdpi.com/article/10.3390/molecules29215192/s1, Table S1. The calculated atomic positions of SrAl_2_O_4_ in fractional coordinates; Figure S1. The variations in lattice parameters (*a*, *b*, *c*) and their ratios (*a*/*a*_0_, *b*/*b*_0_, *c*/*c*_0_) under different pressure conditions..
