# Supplementary material for: Structural, Elastic, Electronic, Dynamic, and Thermal Properties of SrAl2O4 with an Orthorhombic Structure Under Pressure
Source: Molecules. 2024 Nov 2;29(21):5192. doi: 10.3390/molecules29215192 (PMC11547771; doi:10.3390/molecules29215192)
Supplement: Supplementary file 1 [file molecules-29-05192-s001.zip › molecules-3153871-supplementary.pdf]

Structural, Elastic, Electronic, Dynamic, and Thermal Properties of $SrAl_2O_4$ with an Orthorhombic Structure Under Pressure

Hongli Guo [1,*], Huanyin Yang [1], Suihu Dang [1], Shunru Zhang [2] and Haijun Hou [3,*]

1 College of Electronic and Information Engineering, Yangtze Normal University, Chongqing 408000, China; yuxinyin83@163.com (H.Y.); dangsuihu@126.com (S.D.)
2 School of Physics, Electronics and Intelligent Manufacturing, Huaihua University, Huaihua 418008, China; zefer1979@163.com
3 School of Materials Engineering, Yancheng Institute of Technology, Yancheng 224051, China
* Correspondence: 20110022@yznu.edu.cn (H.G.); hhj@ycit.cn (H.H.)

Table of Contents

Table S1 The calculated atomic positions of $SrAl_2O_4$ in fractional coordinates.

Fig. S1 The variations in lattice parameters (a, b, c) and their ratios (a/a_0, b/b_0, c/c_0) under different pressure conditions.

Table S1 The calculated atomic positions of $SrAl_2O_4$ in fractional coordinates.

	Present			Ref.[16]		
Atom	x	*y*	z	x	*y*	z

Sr	0.2430	0.25	0.6571	0.2431	0.25	0.6571
Al1	0.0782	0.25	0.3987	0.0779	0.25	0.3988
Al2	0.5636	0.25	0.6066	0.5635	0.25	0.6065
O1	0.0694	0.25	0.0726	0.0696	0.25	0.0726
O2	0.2988	0.25	0.3562	0.2994	0.25	0.3562
O3	0.3900	0.25	0.0148	0.3902	0.25	0.0149
O4	0.4743	0.25	0.7862	0.4738	0.25	0.7864

Fig. S1 additionally illustrates the lattice parameters a, b, c and their corresponding relative values a/a_0, b/b_0 and c/c_0 under varying pressure conditions (where a_0, b_0, and c_0 denote the corresponding quantities under conditions of zero pressure (P) and zero temperature (T)). It is evident that as pressure increases, there is

a noticeable decline in the values of a, b, c, a/a_0, b/b_0 and c/c_0.

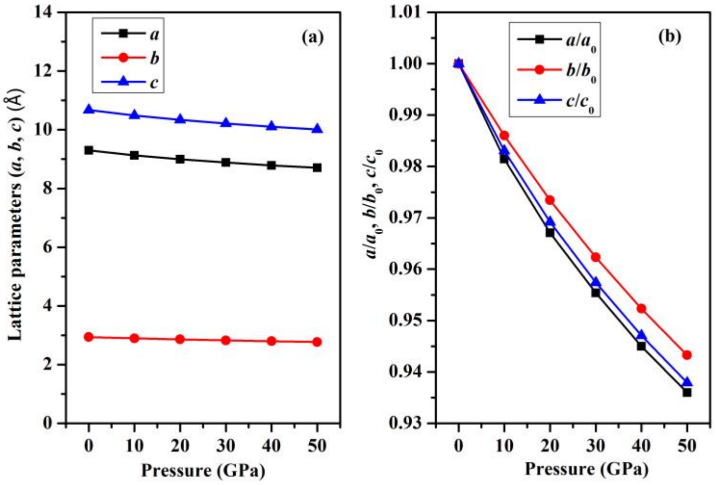

Fig. S1 The variations in lattice parameters (a, b, c) and their ratios (a/a_0, b/b_0, c/c_0) under different pressure conditions.
